# Microwave-assisted synthesis of layered basic zinc acetate nanosheets and their thermal decomposition into nanocrystalline ZnO

**DOI:** 10.1186/1556-276X-9-11

**Published:** 2014-01-08

**Authors:** Afshin Tarat, Chris J Nettle, Daniel T J Bryant, Daniel R Jones, Mark W Penny, Richard A Brown, Ravish Majitha, Kenith E Meissner, Thierry G G Maffeis

**Affiliations:** 1 Multidisciplinary Nanotechnology Centre, College of Engineering, University of Swansea, Singleton Park, Swansea SA28PP, UK; 2 SPECIFIC, College of Engineering, University of Swansea, Baglan, Swansea SA2 8PP, UK; 3 Department of Materials Science and Engineering, Texas A&M University, College Station, TX 77843, USA; 4 Department of Biomedical Engineering, Texas A&M University, Emerging Technology Building, College Station, TX 77843-312, USA

**Keywords:** ZnO, Nanocrystalline, LBZA, Gas sensor, Solar cell

## Abstract

We have developed a low-cost technique using a conventional microwave oven to grow layered basic zinc acetate (LBZA) nanosheets (NSs) from a zinc acetate, zinc nitrate and HMTA solution in only 2 min. The as-grown crystals and their pyrolytic decomposition into ZnO nanocrystalline NSs are characterized by scanning electron microscopy (SEM), energy-dispersive X-ray spectroscopy (EDS), atomic force microscopy (AFM), X-ray diffraction (XRD) and photoluminescence (PL). SEM and AFM measurements show that the LBZA NSs have typical lateral dimensions of 1 to 5 μm and thickness of 20 to 100 nm. Annealing in air from 200°C to 1,000°C results in the formation of ZnO nanocrystalline NSs, with a nanocrystallite size ranging from 16 nm at 200°C to 104 nm at 1,000°C, as determined by SEM. SEM shows evidence of sintering at 600°C. PL shows that the shape of the visible band is greatly affected by the annealing temperature and that the exciton band to defect band intensity ratio is maximum at 400°C and decreases by a factor of 15 after annealing at 600°C. The shape and thickness of the ZnO nanocrystalline NSs are the same as LBZA NSs. This structure provides a high surface-to-volume ratio of interconnected nanoparticles that is favorable for applications requiring high specific area and low resistivity such as gas sensing and dye-sensitized solar cells (DSCs). We show that resistive gas sensors fabricated with the ZnO NSs showed a response of 1.12 and 1.65 to 12.5 ppm and 200 ppm of CO at 350°C in dry air, respectively, and that DSCs also fabricated from the material had an overall efficiency of 1.3%.

**PACS:**

81.07.-b; 62.23.Kn; 61.82.Fk

## Background

ZnO nanomaterials have attracted significant attention over the past 12 years due to a wide direct band gap (3.37 eV), a large exciton binding energy, a large piezoelectric constant and the availability of a vast range of nanostructure shapes [1]. In the last decade, a variety of different techniques have been used to produce ZnO nanoparticles (NPs). Chemical bath synthesis [2] is a widespread method due its simplicity and low temperature. However, it is a lengthy process, requiring hours or even days. Microwave-assisted solution phase growth, with the microwave energy delivered to the chemical precursors through molecular interactions with the electromagnetic field, leads to rapid reactions. ZnO nanostructures have been produced through microwave-assisted growth in minutes, including nanowires and nanosheets (NSs) [3-5], but the microwave-assisted fabrication of layered basic zinc acetate (LBZA) crystals has not been reported. The thermal decomposition of LBZA into ZnO is an efficient route for low-cost mass production of ZnO nanomaterial, especially for applications requiring a high surface-to-volume ratio [6,7]. In a previous publication, we described the growth of LBZA nanobelts and their subsequent decomposition into interconnected ZnO NPs and demonstrated their potential for gas sensing [8]. However, the growth of the LBZA NBs took 20 h, similar to previously reported LBZA growth studies [9,10]. Here, we report on the fabrication of LBZA NSs using a conventional microwave, with the process taking only 2 min. The physical, chemical and optical properties of the LBZA NSs and the ZnO NSs obtained by subsequent air annealing are investigated by scanning electron microscopy (SEM), energy-dispersive X-ray spectroscopy (EDS), atomic force microscopy (AFM), X-ray diffraction (XRD) and photoluminescence (PL). We also demonstrate the promising potential of this novel growth process for practical applications by fabricating and testing gas sensing devices and dye sensitized solar cells (DSCs) using ZnO NPs evolved from the NSs.

## Methods

Without any further purification (purity ≥ 99.0%), 0.1 M Zinc acetate dihydrate (Zn(CH_3_COO)_2_.2H_2_O), 0.02 M zinc nitrate hexahydrate (Zn (NO_3_)_2_.6H_2_O) and 0.02 M Hexamethylenetetramine (HMTA, (CH_2_)_6_ N_4_) from Sigma Aldrich Co. Ltd. (St. Louis, MO, USA) were dissolved in 60 ml deionized water. The resulting solution had a pH of 6.8. It was then placed in a commercial microwave oven at maximum power (800 W, 2,450 MHz) for 2 min. The oven capacity was 25 l and the dimensions of the cavity were 281 × 483 × 390 mm^3^. This resulted in the formation of a white suspension. The structure and morphology of the products were characterized using AFM (NanoWizard® II NanoScience, JPK Instruments, Berlin, Germany), field emission SEM (Hitachi S4800, Hitachi High Technologies, Minato-ku, Tokyo, Japan), XRD (Bruker D8 diffractometer, Billerica, MA, USA) using CuKα radiation and fitted with a LynxEYE detector and photoluminescence (PL) using a He-Cd laser with a wavelength of 325 nm and a Ocean Optics USB2000+ spectrometer (Dunedin, FL, USA), blazed at 500 nm and calibrated using a standard 3,100 K lamp. The excitation power density was approximately 3 mW/mm^2^ for all samples, and the PL spectra were corrected for the detection response of the spectrometer. The PL was performed at room temperature and in air and the XRD diffractogram acquired in θ-2θ mode.

Sample preparation for AFM and SEM consisted of dropcasting a 10-μl droplet of the diluted LBZA NSs suspension on clean silicon wafers followed by drying at 60°C. For the PL characterization, the as-grown product was filtered using a vacuum filtration system. A white thin membrane subsequently formed on the filter paper after drying the product at 60°C for 1 h. The LBZA NSs (either in filtered membrane form or deposited on silicon) were then air annealed in a tube furnace at temperatures from 200°C to 1,000°C for 10 min.

Samples for the resistive gas sensing tests were fabricated by dropcasting 10 μl of the as-grown LBZA suspension onto alumina substrates comprised of a Pt-interdigitated electrode and a Pt track heater at the back. The NSs were left to sediment on to the substrate and form a film for 1 min after which the drop of suspension was removed and the sensor was annealed at 400°C in air for 30 min. The response of the ZnO NSs to CO was measured in dry air using a custom built gas flow apparatus (details are published elsewhere [8]) under a 400-sccm total flow and at 350°C.

To make DSCs, vacuum filtration was used to separate the grown product from the growth solution, adding a 1:1 volume mix of ethanol to deionised water when almost dry. The resulting LBZA NS paste was then spread onto FTO glass using a spatula, following by annealing at 400°C. The DSCs were then fabricated by a method reported elsewhere [11] using a dye solution made up of cis-bis(isothiocyanato)bis(2,2-bipyridyl-4,4-dicarboxylato)-ruthenium(II)bis-tetrabutylammonium2 in a 1:1 volume mix of ethanol to deionised water. The electrolyte solution was 0.1 M LiI, 0.6 M tetrabutyl ammonium iodine (TBAI), 0.5 M 4-tert butylpyridine (4-TBP) and 0.1 M I2 In 3-methoxypropionitrile (MPN). The DSCs were characterized using a PV Measurements QEX10 quantum efficiency measurement system (Boulder, CO, USA) and a Newport Oriel AAA solar simulator (Stratford, CT, USA).

## Results and discussion

**Figure 1:**
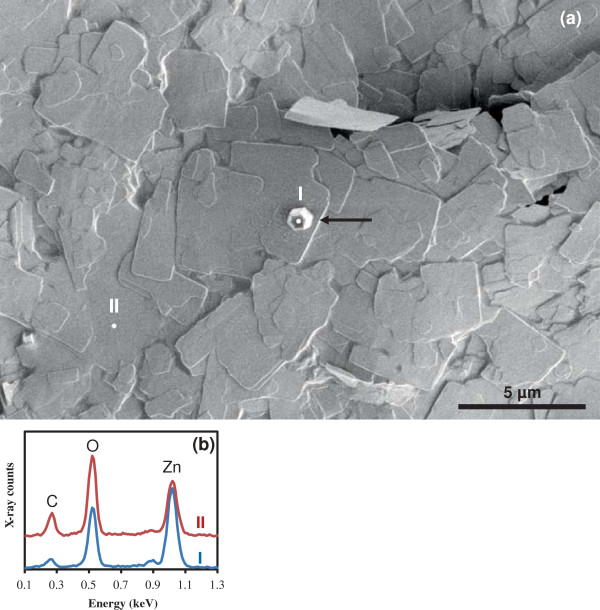
**SEM image of LBZA NSs on Si and typical EDS spectra. (a)** SEM image of LBZA NSs on Si, showing the sheet like morphology of most of the growth as well as a hexagonal crystal (black arrow). The image was acquired at 1 kV without metal coating. The crosses labeled I and II refer to locations similar to where the EDS spectra of **(b)** were acquired. **(b)** Typical EDS spectra corresponding to the locations shown in **(a)**. The EDS spectra were acquired at 3 kV to minimize charging and excitation volume.

Figure 1a shows a SEM image of the typical morphologies of as-synthesized LBZA NSs, displaying the typical lamellar structure of LBZA. The crystals have a rectangular shape with lateral dimensions between 1 and 5 μm. The black arrow on Figure 1 points to a thicker crystal with a different, hexagonal, morphology typical of ZnO. The growth of similar ZnO crystals from zinc acetate solutions has been reported previously [12] and in order to confirm the composition, EDS was performed on the NSs and on the hexagonal crystals. The results are shown in Figure 1b. The spectrum taken from the NSs (red) gives a composition of 23.7% Zn, 57.5% O and 18.8% C, in good agreement with the stoichiometric composition of LBZA of 21.7% Zn, 60.9% O and 17.4% C for Zn_5_(OH)_8_(CH_3_COO)_2_.2H_2_O. On the other hand, the point spectrum taken from the hexagonal crystal (blue) gives a composition of 41% Zn, 50.6% O and 8.4% C, close to what is expected for ZnO. The presence of carbon and the excess oxygen is likely due to the X-ray excitation volume being slightly larger than the crystal itself and therefore including X-rays from the NSs in the spectrum. From many growth runs and SEM imaging, we observed that the occurrence of the ZnO crystals is about one per 0.01 mm^2^ of surface analyzed and therefore they are very rare compared to the NSs.

**Figure 2:**
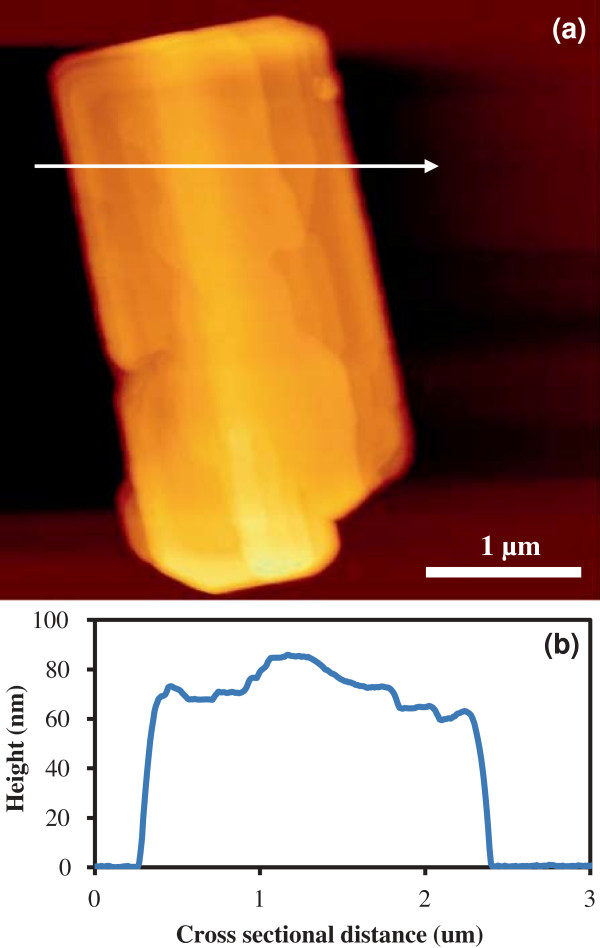
**AFM image of a NS and height profile. (a)** AFM image of a NS acquired in intermittent contact mode. The arrow shows the location of the height profile **(b)**.

For individual NSs, AFM scans yielded heights between 20 and 100 nm. Figure 2 shows an AFM image of typical NS with a height of 85 nm and possessing distinctive surface features, such as steps and terraces, indicative of layer-by-layer growth. The line profile of Figure 2b shows that the heights of the steps vary from 2 to 10 nm.

**Figure 3:**
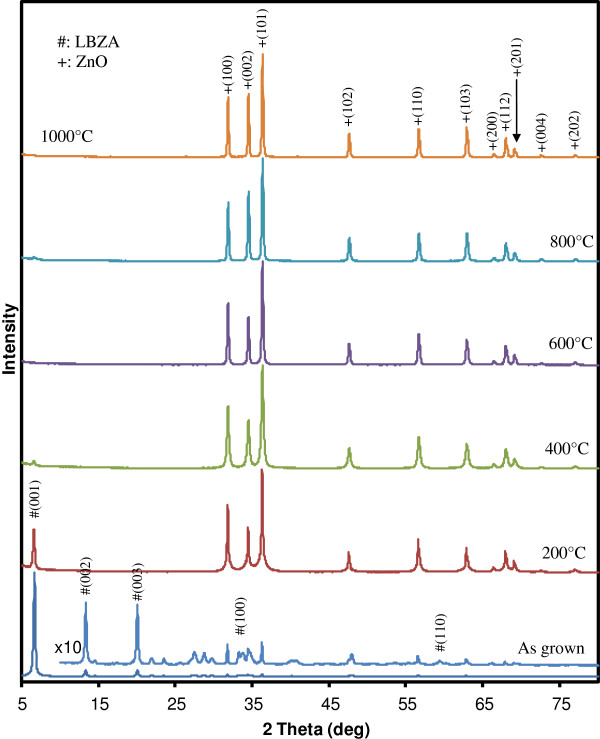
**XRD diffractograms of the as-grown LBZA NSs and following anneals at 200°C, 400°C, 600°C, 800°C and 1,000°C.** The region of the as-grown LBZA diffractogram corresponding to two theta angles greater than 10° has been magnified 10 times.

The XRD diffractograms from the as-synthesized NSs as well as the samples after annealing at increasing temperatures (from 200°C to 1,000°C) are presented in Figure 3. The as-grown NSs show the characteristic main LBZA (001) peak at 6.657°, corresponding to an interplanar spacing within a single layer of 1.32 nm and confirming their composition as Zn_5_(OH)_8_(CH_3_COO)_2_.2H_2_O [6,7,9,12-14]. The (002) and (003) peaks at 13.32° and 20.05° are also visible and the × 10 magnified region reveals further peaks around 33.5° and 59.3° attributed to the (100) and (110) reflections [6,13,14]. The magnified region also shows peaks corresponding to ZnO, possibly coming from the hexagonal crystals discussed earlier. Their small intensity relative to the (001) LBZA peak is in good agreement with the SEM analysis which showed a low occurrence compared to the NSs. Several broad small peaks suggest the presence of a small amount of amorphous phases. Following annealing at 200°C, the ZnO peaks intensity increases whilst the zinc acetate (001) peak is reduced. After annealing at 400°C, the zinc acetate peak decreases further and is barely detectable, confirming the complete decomposition into ZnO. This is in good agreement with previous thermal gravimetric analysis results which reported that the transition to ZnO starts at 150°C but is not fully complete until above 350°C [6]. Annealing at higher temperatures generally increased the intensity of the wurtzite ZnO peaks and decreased their width, indicating an increase in crystallite size with temperature.

**Figure 4:**
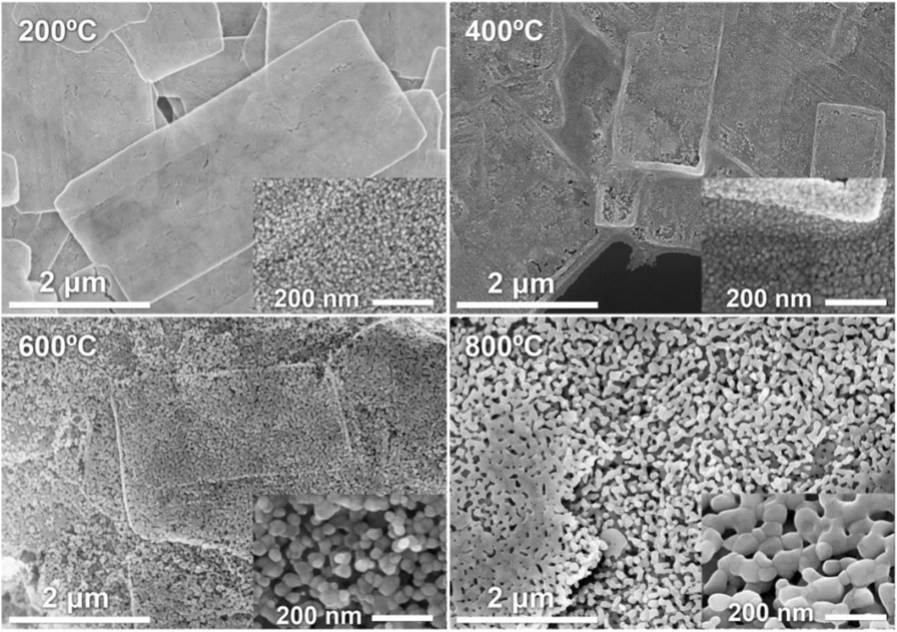
**SEM images from annealed LBZA NSs at 200°C, 400°C, 600°C and 800°C.** Scale bar 2 μm. Insets: detail of the nanocrystals, scale bar 200 nm.

Figure 4 shows SEM images of LBZA NSs after annealing at 200°C, 400°C and 800°C. The 200°C image clearly shows interconnected NPs within the NSs and increasing temperature results in a size increase of the ZnO NPs, confirming the XRD data. The results of the size analysis are given in Table 1 and show that the crystallite size increases from 15.8 nm at 200°C to 104 nm at 1,000°C. In addition, sintering of the NPs is observed at 600°C (Figure 4) After annealing at 800°C, the sintering process intensifies. The NSs keep their shape and their structures reasonably constant even after the 1,000°C anneal, similar to previous results for nanobelts [8]. The thickness of the NSs was not significantly altered by the annealing process.

**Table 1:** SEM size measurement of the crystallite size for ZnO NSs evolved from LBZA NSs annealed at different temperatures and their standard deviation

**Temperature (°C)**	**200**	**400**	**600**	**800**	**1,000**
Average size (nm)	15.8	23.1	37.4	70.3	104
Standard deviation (nm)	3.2	9.34	14.66	22.6	38.5

**Figure 5:**
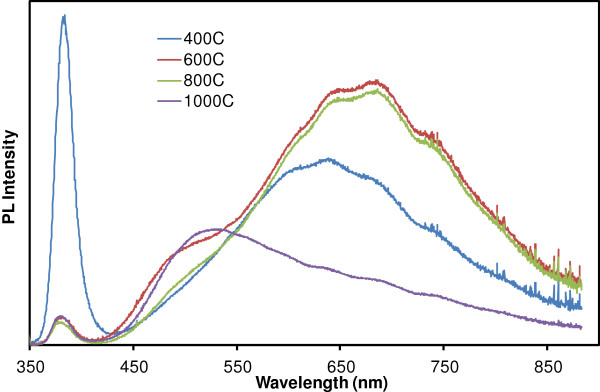
**PL spectra of ZnO NSs produced via annealing of LBZA NSs in air at 400°C, 600°C, 800°C and 1,000°C.** The excitation wavelength was 325 nm and the power density was approximately 3 mW/mm^2^ for all samples.

Figure 5 shows the PL spectra acquired from ZnO NSs produced by annealing of LBZA NSs at various temperatures in air. The spectra show the narrow near band edge (NBE) peak at 380 nm and the broad visible band typical of ZnO, associated with deep level emission (DLE). The DLE band is centered around 630 nm for the NSs produced at 400°C, resulting in a red orange emission, which is significantly red-shifted compared to the green/yellow emission typical of single crystal ZnO nanostructures such as nanorods [15] and tetrapods [16]. After annealing at 600°C and 800°C, the band broadens and the orange contribution of the visible band becomes more intense. Annealing at 1,000°C resulted in a predominantly green visible band. The DLE contribution is conventionally attributed to oxygen vacancies and other bulk lattice defects, despite evidence pointing to surface defects for nanostructures [17,18]. Our results show that the NBE to DLE band ratios, calculated from the area under the PL spectra, are 0.161 at 400°C, 0.011 at 600°C, 0.009 at 800°C and 0.024 at 1,000°C. As the nanoparticle size within the NSs increases with temperature, the surface-to-volume ratio decreases, therefore indicating that the DLE is not caused by surface effects in our case. It would instead point towards a decrease in optical crystal quality at annealing temperatures higher than 400°C. Hsieh et al. [19] have reported a large DLE band for their thin ZnO films after annealing at 900°C in air compared to annealing in vacuum or pure oxygen. They attributed the DLE to increased oxygen vacancies. However, Djusiric et al. [17,18,20] have written extensively about the origin of the DLE band and, according to their findings, the strong orange red component we observe at 400°C, 600°C and 800°C could be caused by zinc interstitials, and/or excess oxygen. They also observed a large increase in the orange/red part of the DLE band and a decrease in the NBE intensity after annealing their samples in air at 600°C, similar to what we report here. The predominance of green emission in the DLE after annealing at 1,000°C could be caused by increased recombination at grain boundaries. Figure 5 clearly shows several individual components, corresponding to different radiative transitions, which vary in intensity with the annealing temperature. Further investigations of this material system could therefore help shed light on the origin of the visible band.

**Figure 6:**
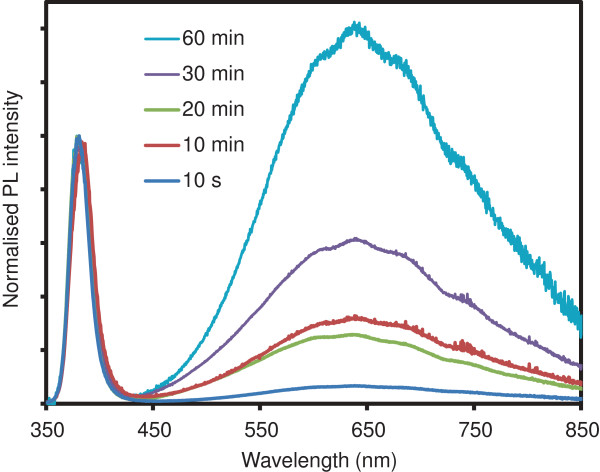
**PL spectra of ZnO NSs produced via annealing of LBZA NSs in air at 400°C.** The NSs were annealed for 10 s, 10 min, 20 min, 30 min and 60 min. The spectra were normalized to intensity of the NBE band. The excitation wavelength was 325 nm and the power density was approximately 3 mW/mm^2^ for all samples.

We also investigated the effect of annealing time on the PL properties. Figure 6 shows spectra normalized to the NBE intensity taken from samples annealed in air at 400°C for 10 s, 10 min, 20 min, 30 min and 60 min. The 10 s sample was removed from the furnace within 10 s after the furnace reached the 400°C setpoint and left to cool down at room temperature. The other samples were removed from the furnace after a given time and left to cool down in the same manner. Figure 6 shows that the intensity of the NBE band decreases relative to the DLE band with increasing temperature. This is particularly noticeable between the samples that were annealed for 10 s and 60 min, where the NBE to DLE ratio decreases from 1.329 to 0.073. The 10- and 20-min anneals result in very similar spectra (ratios of 0.316 and 0.361, respectively), whilst the 30 min sample shows a slight decrease in the ratio (0.155). It should be noted that the 10-, 20- and 30-min spectra are within the variability observed from different growth batches, where environmental conditions such as ambient humidity at the time of synthesis, anneal and measurement might affect the intensity ratio. This also explains the difference in ratio for the 400°C, 10-min spectra in Figures 5 and 6. However, the difference between the 10-s and 60-min sample is significant. The shape of the DLE band remains the same, which points towards a decrease in the probability of band-to-band recombination, rather than an increase in the concentration of a specific defect. Further work is underway to investigate this effect. SEM analysis showed an increase in particle size with increasing annealing time, from 22 nm for the 10-s sample to 32 nm for the 60-min sample.

**Figure 7:**
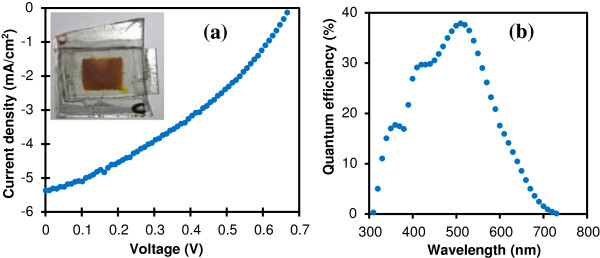
**Performance of a 1-cm**^**2 **^**DSC fabricated with ZnO NSs. (a)** Current–voltage curve of the DSC recorded under one sun illumination, yielding a short circuit current density of 5.38 mA/cm^2^, an open circuit voltage of 0.67 V and a fill factor of 35.6%. The inset shows the DSC. The NSs were produced by annealing LBZA NSs at 400°C. **(b)** The incident photon to charge carrier efficiency as a function of wavelength for the cell.

In order to assess the potential of the microwave-assisted LBZA synthesis process for practical ZnO applications, we fabricated DSCs using the ZnO NSs produced by air annealing the LBZA NSs at 400°C in air to replace the traditional TiO_2_ NP scaffold. Figure 7a shows the current voltage characteristics of a DSC under one sun illumination. The open circuit voltage, short circuit current density and fill factor were 0.67 V, 5.38 mA/cm^2^ and 35.6%, respectively. The quantum efficiency (incident photon to charge carrier efficiency) as a function of wavelength is shown on Figure 7b. The characteristic dye absorption peaks can be seen at 410 and 525 nm, as well as the ZnO band edge absorption at 370 nm. The overall efficiency was 1.3%, better than some previously reported ZnO nanowire DSCs [21] and compares well cells made with very high aspect ratio ZnO NWs (1.5%) [22] but still lower than cells based on hierarchical ZnO, where the high surface-to-volume ratio led to efficiencies of 2.63% [23]. It should be noted that the thickness of the ZnO NSs film could not be controlled accurately in this initial experiment, resulting in varying degree of dye loading. In the future, we look to improve the efficiency by optimizing the thickness and exploring different dyes.

**Figure 8:**
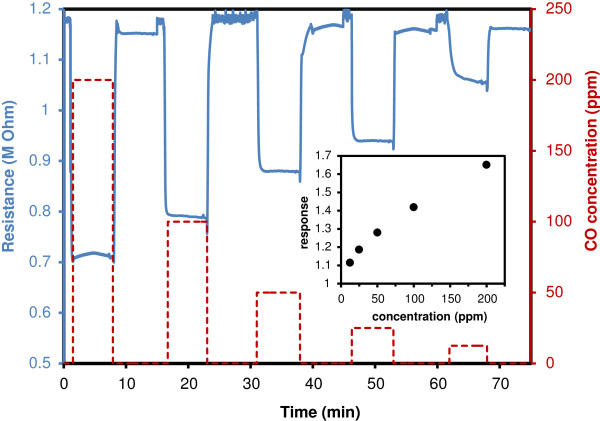
**Resistance response to CO of a film of ZnO NSs at 350°C.** The blue solid line shows the resistance versus time curve as various CO concentrations are mixed with the flowing dry air of the test chamber. The decreasing CO concentrations, from 200 to 12.5 ppm, are shown by the dashed red line. The inset shows the response of the sensing film as a function of CO concentration.

We also fabricated resistive gas sensing devices using the same material with Figure 8 showing the effect of CO exposure on the resistance of a film of ZnO NSs obtained by annealing LBZA NSs at 400°C. The graph shows that the response, defined as *R*(air)/*R*(CO), was 1.65, 1.48, 1.32, 1.22 and 1.13 at 200, 100, 50, 25 and 12.5 ppm of CO, respectively. The response time was under 30 s for 100 ppm, whilst the recovery time was 40 s. Figure 8 demonstrates the stability of the sensing and highlights the potential of the material for this application. The sensitivity could be improved further by optimization of the thickness and cohesion of the films using organic binders.

## Conclusion

We report a novel technique for the production of ZnO nanocrystalline NSs through thermal decomposition of LBZA NSs. The LBZA NSs were produced by a low-cost, high-yield and low-temperature microwave-assisted aqueous technique in only 2 min. The NSs are mostly rectangular in shape with sides of 1 to 5 μm and a minimum thickness of 20 nm, with a structure typical of lamellar growth. Partial thermal decomposition into ZnO occurs after annealing in air at 200°C and is complete after 400°C, producing ZnO nanocrystalline NSs. Annealing at higher temperatures results in an increase of the nanoparticle size within the NSs and sintering was observed after 600°C. The NSs keep their shape even after annealing at 1,000°C. PL data show a significant deep level emission comprising several distinct transitions. The exciton to deep level intensity ratio was highest at 400°C and decreased at higher temperatures and with longer annealing times at 400°C. The shape of the deep level band was also altered by the annealing temperature. ZnO NSs produced by annealing at 400°C were used to fabricate DSCs and resistive gas sensors. The DSCs showed an overall efficiency of 1.3% whilst the response of the sensors at 350°C was 1.65 and 1.13 at 200 and 12.5 ppm, respectively. These results highlight the potential of the material for device applications.

## Abbreviations

DLE: Deep level emission; DSC: Dye-sensitized solar cells; EDS: Energy-dispersive X-ray spectroscopy; HMTA: Hexamethylenetetramine; IPCE: Incident photon to charge carrier efficiency; LBZA: Layered basic zinc acetate; NBE: Near band edge; NPs: Nanoparticles; NSs: Nanosheets; PL: Photoluminescence; SEM: Scanning electron microscopy; XRD: X-ray diffraction.

## Competing interests

The authors declare that they have no competing interests.

## Authors’ contributions

AT synthesized all the LBZA and ZnO material, conducted the SEM and AFM characterization, measured the gas sensing properties and co-wrote the paper with TGGM. DRJ, CJN and DTJB fabricated and characterized the solar cells. RAB and MWP contributed to the gas sensing measurement optimization and the size analysis. KEM and RM conducted the XRD analysis. TGGM designed the experiments and co-wrote the manuscript. All authors have read and approved the final manuscript.
